# Evaluation of the anatomical parameters for normal tissue sparing in the prone position radiotherapy with small sized left breasts

**DOI:** 10.18632/oncotarget.12662

**Published:** 2016-10-14

**Authors:** Hyunjung Kim, Jinyoung Kim

**Affiliations:** ^1^ Department of Radiation Oncology, Inje University Haeundae Paik Hospital, Busan, Korea; ^2^ Department of Radiation Oncology, Yonsei University College of Medicine, Seoul, Korea

**Keywords:** breast cancer, prone position, small breast, radiotherapy, heart saving

## Abstract

Prone position radiotherapy for a small (< 750 cm^3^) breast is controversial because of the variable benefits for the irradiated heart volume. The objective anatomical parameters related with chest wall shape that can determine the heart dose sparing patients in the prone position.

Twenty-one patients underwent CT-simulation in supine and prone position. Dose volume parameters were compared and the objective indexes such as the Haller index, anthropometric index, mid-sternum thickness, and central lung distance (CLD) were evaluated the relationship between the shape of the chest wall and irradiated normal tissue volume in prone position.

The median breast volume was 440.10 cm^3^ (range, 151.5–727.41 cm^3^). There was no difference of breast target volume between supine and prone position (*p* = 0.178). The Haller index under 2.5 (*p* = 0.046), an anthropometric index over 0.05 (*p* = 0.007), and the CLD over 2 (*p* = 0.023) conferred a greater heart sparing effect in the prone position.

In conclusions, the objective anatomical parameters related chest wall shape predict the decrease in irradiated heart volume in the prone position. Therefore, it is possible to screen for patients with a reduced heart volume irradiation among those with small breasts before applying prone position radiotherapy.

## INTRODUCTION

In early stage breast cancer, there is no significant difference in survival or local control rate between mastectomy and breast-conserving surgery (BCS) followed by radiotherapy [1–3]. Radiotherapy after BCS generally treats the whole breast using a tangential technique with two fields of radiation beams in the supine position. This method includes the entire breast parenchyma, but it is also irradiates other tissues: the skin, ipsilateral lung, heart, and contralateral breast. Therefore, radiation-induced morbidity can occur [4, 5].

There are some options to reduce radiation-induced morbidity. First, intensity modulated radiotherapy (IMRT) techniques using volumetric arc therapy (V-MAT) or static tomotherapy can reduce the radiation dose to other tissues [6, 7]. Second, respiration control including the deep inhalation breath holding (DIBH) technique can reduce the irradiated heart volume [8, 9]. Third, the prone position can reduce the radiation dose to the normal tissue volume [10, 11]. The prone position makes breast tissue unfold away from body tissues because of gravity, and therefore, radiation homogeneity and conformity might be increased [12]. Increased radiation dose homogeneity and conformity leads to a reduction in the radiation dose to tissues such as the lung and heart [13], [14]. Therefore, a patient with large and pendulous breasts has an advantage in the prone position.[15, 16]

However, prone position radiotherapy for a small or thin breast is controversial. This is because these patients have no advantage from gravity in the prone position because of their small and dense breast parenchymal tissue. It has been reported [17] that shortening the distance between the heart and chest wall because of gravity might increase the irradiated volume of the heart in the prone position. Radiation dose homogeneity and conformity worsens as the breast parenchymal volume decreases [18]. There are few studies of prone position radiotherapy in patients with small breasts. But most of Asian women and leaner body shaped women had a small sized breast.

In this study, we hypothesized that there is a relationship between irradiated normal tissue volume and the chest wall shape rather than the breast shape and volume. We analyzed several objective anatomical parameters that might be able to predict the value of sparing other tissues in patients with small sized breasts in the prone position during radiation therapy.

## RESULTS

### Patients and treatment characteristics

There were 21 enrolled women with a mean age of 54 (range, 38–80). All patients had left breast cancer with a breast volume of less than 750 cm^3^. Three cases were stage 0 and 18 were stage IA. Moreover, patients had ductal carcinoma (*n* = 2), lobular carcinoma *in situ* (*n* = 1), and invasive ductal carcinoma (*n* = 12), invasive lobular carcinoma (*n* = 2), mucinous carcinoma (*n* = 2), and tubular carcinoma (*n* = 2). Their body mass index (BMI) was median 23.43 (range, 19.28–30.23).

The patients’ anatomical parameters in supine position are summarized in Table 1. The median breast volume was 440.10 cm^3^ (range, 151.5–727.41) in the prone position, and the breast volumes were not significantly different when compared to in the supine position (*p* = 0.178). The patients were divided into groups based on breast volume < 350 cm^3^, 351–550 cm^3^, 551–750 cm^3^, and there were 7, 6, and 8 per group, respectively. The median Haller index was 2.63 (range, 1.86–3.37), the median anthropometric index was 0.05 (range, 0.002–0.1), and the median midsternum thickness was 1.69 (range, 0.79–2.47). The median central lung distance (CLD) for radiation treatment plans in the supine position was 2.37 (range, 1.28–3.17).

**Table 1:** Patients anatomical characteristics in supine position

	*N* = 21	(%)
Breast volume (cm^3^) in prone		
Median	440.1	
Range	(151.5–727.41)	
≤ 350	7	(33.34)
351–550	6	(28.57)
551–750	8	(38.09)
BMI (kg/m^2^)		
Median	23.43	
Range	(19.28–30.23)	
Haller index		
Median	2.63	
Range	(1.86–3.37)	
Anthropometric index		
Median	0.058	
Range	(0.002–0.1)	
Mid sternum thickness (cm)		
Median	1.69	
Range	(0.79–2.47)	
CLD (cm)		
Median	2.37	
Range	(1.28–3.17)	

CLD, central lung distance.

### Planning values

Table 2 shows the dosimetric endpoints of radiation in the supine and prone position. Whereas conformity number (CN) has statistically different according to position (*p* < 0.0001), there was no difference in terms of homogeneity index (HI) (*p* = 0.079). The lung volume was approximately 11% higher when measured in the prone position than in the supine position (*p* < 0.0001). In the supine position, the irradiated ipsilateral lung volume was 136 cm^3^, compared to 17.02 cm^3^ in the prone position (*p* < 0.0001). The V20 (*p* = 0.001), V5 (*p* = 0.001), and D_mean_ (*p* = 0.001) for the heart were significantly different. However, the left anterior descending coronary artery (LAD) demonstrated no significant differences in V40 (*p* = 0.148), V5 (*p* = 0.870), D_mean_ (*p* = 0.372), or D_max_ (*p* = 0.663).

**Table 2:** Dosimetric endpoints of radiation in the supine and prone position

	prone setup	supine setup			*p*-value
		(SD)		(SD)	
Breast					
Volume (cm^2^)	440.10	155.93	445.04	156.03	0.178
HI	1.12	0.03	1.10	0.03	0.079
CN	0.76	0.06	0.71	0.53	0.000
V105 (%)	1.07	1.50	1.34	1.33	0.478
V107 (%)	0.00	0.00	0.00	0.00	
Ipsilateral lung					
Volume (cm^3^)	1114.15	177.97	999.79	153.09	0.000
V30 (%)	1.16	1.66	12.80	2.98	0.000
V20 (%)	1.48	1.85	14.11	3.04	0.000
V10 (%)	2.24	2.35	17.98	3.24	0.000
V5 (%)	3.95	3.31	25.33	3.71	0.000
D_mean_ (Gy)	1.46	0.98	7.92	1.42	0.000
D_max_ (Gy)	42.31	12.19	50.30	0.76	0.000
In-field lung volume	14.28	17.02	136.55	42.33	0.000
Heart					
V20 (%)	3.44	2.54	6.28	3.39	0.001
V5 (%)	7.35	3.67	10.65	4.45	0.001
D_mean_ (Gy)	2.76	1.22	4.14	1.64	0.001
D_max_ (Gy)	49.21	2.22	50.04	3.56	0.370
LAD					
V40 (%)	16.93	13.92	22.62	15.42	0.148
V5 (%)	42.48	16.19	43.16	13.04	0.870
_mean_ (Gy)	13.47	6.88	15.08	6.71	0.372
D_max_ (Gy)	42.68	10.78	43.89	10.41	0.663

SD, standard deviation; HI, Homogeneity index; CN, Conformity number; LAD, left anterior descending coronary artery.

### Effects of anatomical variables

Table 3 shows that the mean differences of irradiated lung, heart and LAD volumes classified by anatomical variables. The patients were divided into three groups by breast volume: < 350 cm^3^, 351–550 cm^3^, and 551–750 cm^3^. The differences in irradiated lung volume between the supine and prone position for these three groups were 141.85 cm^3^, 126.27 cm^3^, and 96.72 cm^3^, respectively. In fact, the CLD value greater than 2 obtained after the radiation treatment plan that was established in the supine position shows that larger lung volume values can be spared in the prone position.

The irradiated heart volume was different in the supine position compared with the prone position when the breast volume was 551–750 cm^3^ (*p* = 0.008). The thick mid sternum group (*p* = 0.009) can more heart sparing in the prone position. When the BMI was < 23.4, there was a significant difference between the prone and supine groups (*p* = 0.005). The Haller index showed a significant difference in terms of heart sparing when greater than 2.5 in prone position. An anthropometric index over 0.05 had a greater heart sparing effect in the prone position than in the supine position. A large CLD resulted in a greater heart sparing effect in the prone position. However, for LAD, there was no difference based on the chest wall shape indexes.

## DISCUSSION AND CONCLUSIONS

Prone positioned radiotherapy has a benefit in terms of irradiated lung volume in all cases, but the prone position for small breasts is not actively used because of the variable irradiated heart and LAD volume [17], [19]. In this study, prone position radiotherapy minimized the irradiated lung volume, but there was no advantage for the LAD. Compared with the supine position, the distance between the left ventricle and anterior rib is shortened by gravity [12].

Formenti et al. [19] showed that they divided into groups based on breast volume < 750, 751–1500, >1500 cm^3^. They insisted that there was a patient with large breasts (over 750 cm^3^) has an advantage in the prone position. But prone position for a small (under 750 cm^3^) or thin breast is controversial. But most of Asian women and leaner body shaped women had a small sized breast. Even 550–750 cm^3^ sized breast consider large breast volume in Asian patients. Therefore we divided into groups based on breast volume < 350, 351–750, > 750 cm^3^.

Chen et al. [7] reported that there were no significant differences between the prone and supine position for irradiated heart volume based on a breast volume of 450 cm^3^. On the contrary, our study showed that a decreasing irradiated heart volume was obtained in the prone position for the largest breast volume group (551–750 cm^3^). Our results demonstrate that even though the breast volume is small, the benefit to the irradiated heart volume depends on how the volumetric criteria are applied. Varga et al. [20, 21] reported that patients with a BMI greater than 26.3 benefit from heart protection in the prone position. In this study, however, the patients’ median BMI was 23.43 (range, 19.28–30.23), lower than that of the patients in Varga's study. Heart protective effects were also observed in our study in patients with a BMI less than 23.43. Because of ethnic differences, BMI-based results might be different between Asians and western populations. However, breast volume and BMI are quantitative numerical values; therefore, it is difficult to predict the benefits of irradiated heart volume in the prone position.

In this study, we applied various anatomical indexes to objectively predict the decrease in irradiated heart volume in the prone position. We found that the reduced irradiated heart volume was significantly larger in the group with a Haller index less than 2.5 (*p* = 0.0046), the group with an anthropometric index over 0.05 (*p* = 0.007), and the group with a CLD over 2 (*p* = 0.023). Because these indexes reflect the chest wall shape, it possible that these three indexes might be able to predict the heart sparing benefits in certain conditions.

The smaller the heart dose and volume patients receive, the lower the incidence of cardiovascular disease [20]. If the chest wall shape of patients is expressed by an objective numerical value, it is possible to screen for patients with a reduced heart volume irradiation among those with small breasts before applying prone position radiotherapy. Accordingly, before generating treatment plans, it will be possible to consider prone position radiotherapy. However, this study is not free from selection bias because of the small number of patients and the fact that it is a single institution study.

In conclusion, the irradiated heart volume depends on the chest wall shape in women with small breasts. It is possible to predict the reduced irradiated heart volumes in these patients with objective indexes.

## MATERIALS AND METHODS

This study has been approved by the Haeundae Paik Hospital Institutional Review Board.

### Patient positioning and image acquisition

From May to June 2015, 21 early stage left breast cancer patients who underwent BCS and received radiotherapy were enrolled. Simulation computed tomography (CT) images (Somatom Sensation Open, Siemens, Forchheim, Germany) were taken with a thickness of 3 mm with no contrast. The scanning range was from the first cervical to the 12th thoracic spine level. All of the patients had their palpable breast tissue marked by a radio-opaque wire in the supine position. They were re-scanned again in the prone position using a prone breast support (kVue^™^ Access 360^™^, Qfix, Avondale, USA). The position of the knees, arms, and the orientation of nipples and faces were thoroughly recorded. CT scans for the prone position were performed the same as in the supine position.

### Target and non-target tissue delineation

The clinical target volume (CTV) included all visible or palpable breast tissue on simulation CT, followed by the usual guideline to be set from the skin under 5 mm in the supine position [22, 23]. Because of the lack of delineation guidelines for the prone position, the usual delineation guidelines for the supine position were used after considering the changes in anatomy in the prone position. The CTV was delineated by including all visible breast parenchymal tissue, laterally the lateral thoracic artery, medially the lateral position of the sternum, cranially a 2-cm margin from the breast parenchymal tissue, caudally the breast fold, ventrally a 5-mm margin from the skin, and dorsally above the intercostal muscle. The LAD was inferentially delineated from the course of the anterior-interventricular groove. Ipsilateral-lung, contralateral breast and heart, and humeral head were outlined.

**Table 3:** Subgroup analysis of mean differences in the irradiated lung, heart, and left anterior descending coronary artery in the supine and the prone position

		In-field Lung Volume, Mean (± SD) cm^3^					In-field Heart Volume, Mean (± SD), cm^3^					In-field LAD Volume Mean (± SD), cm^3^				
	**No**.	**supine**	**(SD)**	**prone**	**(SD)**	***p*-value**	**supine**	**(SD)**	**prone**	**(SD)**	***p*-value**	**supine**	**(SD)**	**prone**	**(SD)**	***p*-value**
Breast volume (cm^3^)																
≤ 350	7	167.22	37.62	25.37	21.56	0.001	10.15	3.00	8.40	2.64	0.122	44.62	7.34	41.72	12.06	0.516
351–550	6	140.86	35.06	14.59	15.27	0.000	10.23	4.86	7.60	4.68	0.145	39.40	16.15	43.91	21.98	0.577
551–750	8	99.43	31.12	2.72	1.48	0.001	11.77	5.51	5.92	2.80	0.008	47.33	12.63	41.08	11.09	0.460
BMI (kg/m2)																
< 23.4	11	150.72	47.04	19.35	18.34	0.000	9.78	3.10	6.56	3.20	0.005	44.83	12.85	38.15	13.05	0.052
≥ 23.4	10	120.97	31.74	8.69	14.29	0.000	11.60	5.60	8.21	4.11	0.070	41.32	13.67	47.24	18.57	0.460
Mid sternum thickness (cm)																
< 1.6	8	150.40	49.88	19.41	19.13	0.000	9.09	2.70	7.14	3.80	0.039	36.23	10.82	40.04	16.48	0.496
≥ 1.6	13	128.03	36.44	11.12	15.53	0.000	11.61	5.11	7.48	3.73	0.009	47.42	12.77	43.98	16.49	0.565
Haller index																
≤ 2.5	6	143.45	43.49	11.32	12.66	0.001	14.97	3.83	9.43	2.74	0.046	50.57	14.29	55.95	12.17	0.540
> 2.5	15	133.79	43.07	15.46	18.75	0.000	8.92	3.44	6.51	3.73	0.527	40.19	11.70	37.09	14.58	0.527
Anthropometric index																
< 0.05	9	117.09	39.44	12.63	12.04	0.000	8.54	3.94	6.72	3.34	0.092	40.76	12.12	41.51	17.06	0.932
≥ 0.05	12	151.15	39.80	15.51	20.43	0.000	12.23	4.28	7.82	3.97	0.007	44.96	13.92	43.20	16.23	0.645
CLD (cm)																
< 2	4	97.20	26.24	8.13	13.93	0.002	8.60	5.10	5.15	0.79	0.307	47.30	16.58	42.00	19.23	0.775
2–2.49	7	128.91	33.65	19.71	21.83	0.000	9.30	3.73	7.24	3.34	0.023	39.16	10.44	37.09	13.54	0.772
≥ 2.5	10	157.64	42.06	12.93	14.82	0.000	12.41	4.40	8.30	4.37	0.023	44.30	13.85	46.44	17.23	0.617

SD, standard deviation; LAD, left anterior descending artery, BMI, body mass index; CLD, central lung distance.

### Anatomical parameters

The following four indexes were used to measure the chest wall shape in supine position (Table 1)

**Figure 1:**
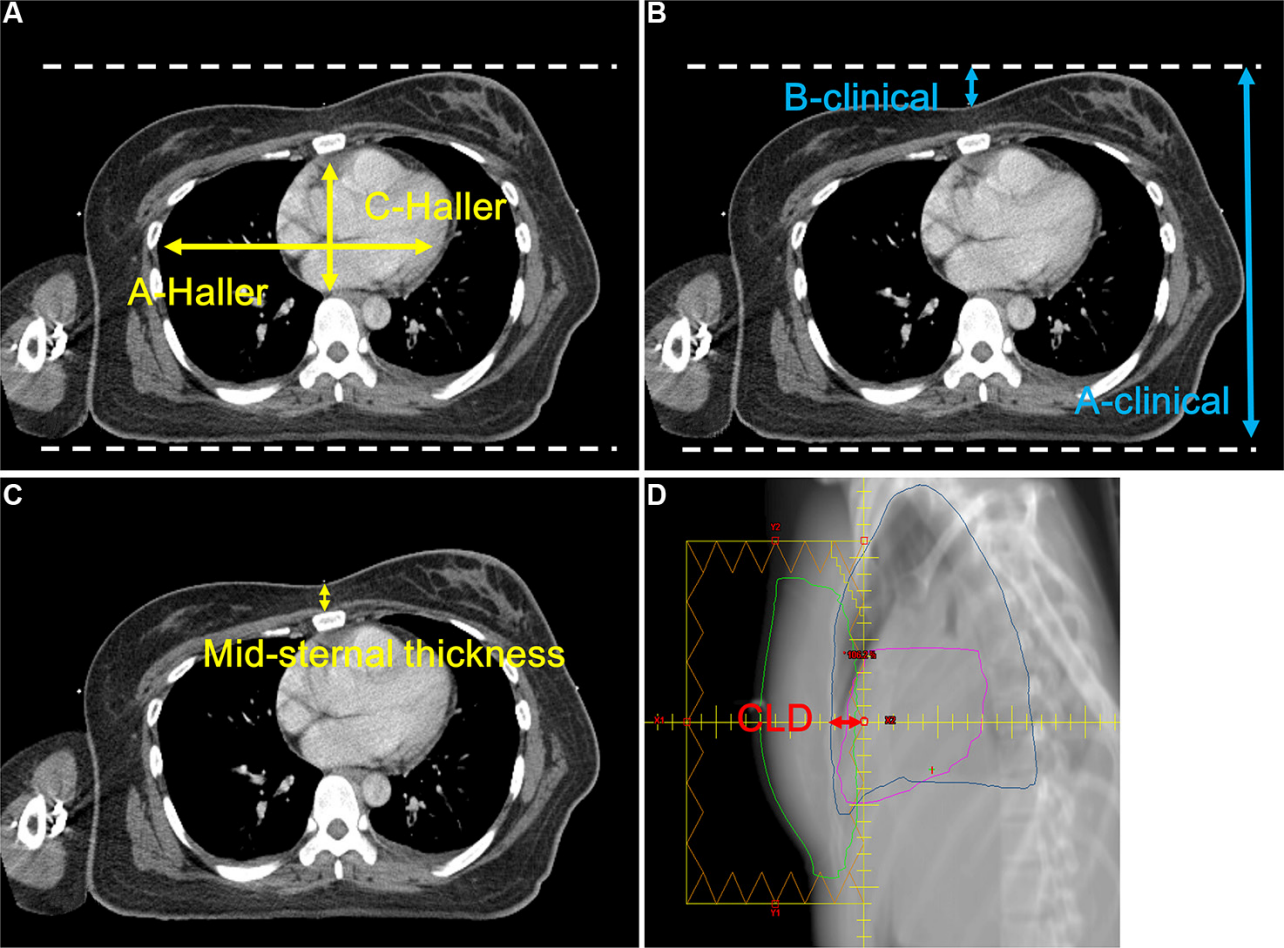
Anatomical parameters. (**A**) Haller index, (**B**) anthropometric index, (**C**) mid sternum thickness, (**D**) central lung distance

1) Haller index [24] (Figure 1A): A CT tomographic measurement for the ratio of the greatest latero-lateral distance (A-Haller) and the shortest sterno-posterior distance (C-Haller) (Haller index = A-Haller/C-Haller). This is an index to evaluate the chest wall deformity of the pectus excavatum, using 2.5 as a normal reference. An index greater than 2.5 indicates that the chest wall is concave.

2) Anthropometric index (Figure 1B): The ratio of the largest anteroposterior diameter (A-clinical) in the distal third area of the sternum level and the largest depth during a deep inhalation (B-clinical) of the same level (anthropometric index = B-clinical/A-clinical). It is a cosmetic indicator used in orthopedics to describe the chest wall shape when viewed from the outside. For women, the chest wall shape is covered with breast parenchyma tissue, and by using the anthropometric index, it is possible to know both the shape of the entire chest wall and breast.

3) Midsternum thickness (Figure 1C): The thickness of the midsternal area at the nipple level in the supine position.

4) CLD (Figure 1D): When planned in the supine position, it is the lung distance in the projection of the tangential fields at the level of the central axis. CLD presents the chest wall concavity in the supine position.

### Dose prescription and radiotherapy planning

**Figure 2:**
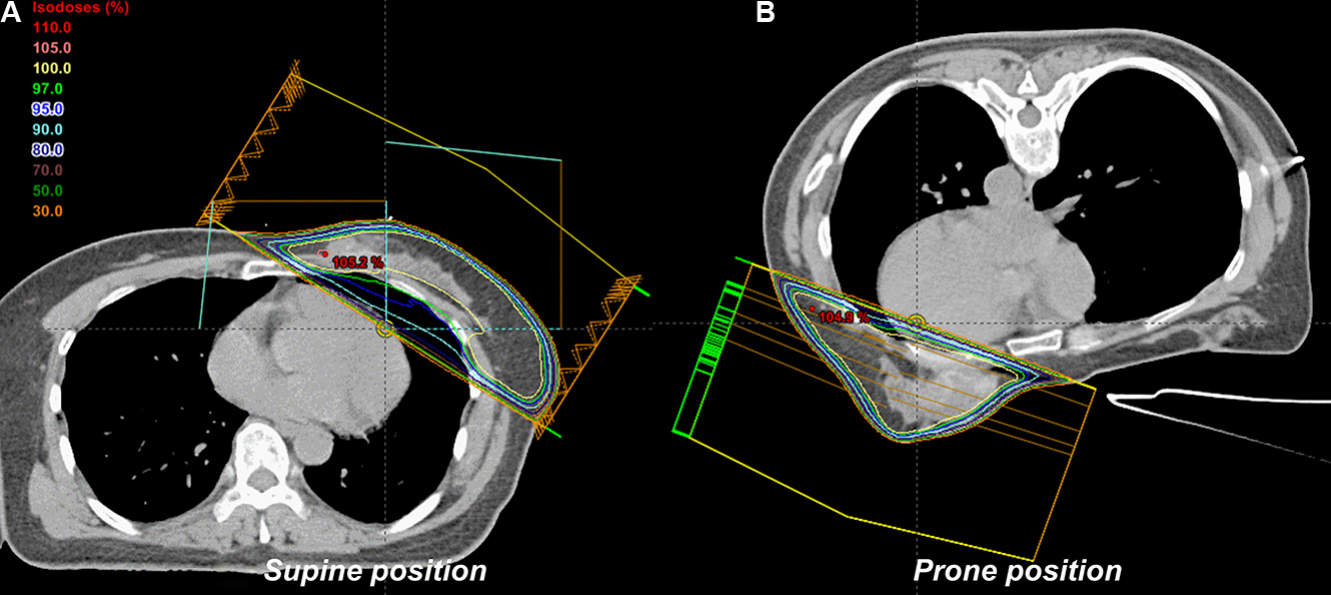
Radiotherapy plan in the (A) supine and (B) prone position

We prescribed 50.4 Gy in 28 fractions over 6 weeks (6–15 MV photons) to the 95% isodose. For each position, standard opposed tangential-fields were employed. Plans were generated on the basis of the International Commission on Radiation Units and Measures (ICRU) dose-homogeneity criteria. They were reviewed with dose distributions and dose-volume-histograms (DVHs). A radiation treatment planning system (Eclipse 11, Varian Medical Systems, Palo Alto, USA) was utilized. The field-in-field (FIF) technique, including of two main fields and four to six subfields, was used to increase dose homogeneity (Figure 2). All of the added subfields were merged into two tangential radiation fields. Moreover, collimator angles were adjusted to the CTV shapes, and asymmetric jaws were optimized to reduce the radiation dose to the lungs. For the radiation dose calculations, a 2.5-mm grid size and an anisotropic analytical algorithm was applied to all plans.

### Planning evaluation

Several dosimetrical parameters such as CN, HI, radiation dose received, and maximum and mean doses were used. The following were used: CN [18]: TV_RI_/TV × TV_RI_/V_RI_ (TV = target volume, TV_RI_ = target volume covered by the reference dose, V_RI_ = volume of the reference dose); HI [25]: D_5%_/D_95%_ (D_5%_ = dose received by 5%, D_95%_ = dose received by 95%). CTV coverages were analyzed by using the volume receiving at least 95% (V95), 105% (V105), and 107% (V107) of the prescribed dose. Lung, heart, and LAD were analyzed by maximum dose (D_max_) and mean dose (D_mean_), V5, V10, V20, and V40.

### Statistical analysis

Statistical analysis was performed with Student's *t*-test, defining a *p* value < 0.05 as statistically significant. All data were then pooled by breast volume into tertiles (< 350, 351–550, and 551–750 cm^3^), BMI, Haller index, anthropometric index, mid-sternum thickness, and CLD. For both the supine and prone position, the differences in the radiation doses to the lung, heart, and LAD volume were calculated and compared by tertile using the Wilcoxon test.
